# The Role of Minor Pilins in Assembly and Function of the Competence Pilus of *Streptococcus pneumoniae*

**DOI:** 10.3389/fcimb.2021.808601

**Published:** 2021-12-22

**Authors:** Vitor Oliveira, Marie-Stephanie Aschtgen, Anke van Erp, Birgitta Henriques-Normark, Sandra Muschiol

**Affiliations:** ^1^ Department of Microbiology, Tumor and Cell Biology, Karolinska Institutet, Stockholm, Sweden; ^2^ Department of Clinical Microbiology, Karolinska University Hospital, Stockholm, Sweden

**Keywords:** competence pilus, minor pilins, natural transformation, *Streptococcus pneumoniae*, type IV pili (T4P), DNA uptake

## Abstract

The remarkable genomic plasticity of *Streptococcus pneumoniae* largely depends on its ability to undergo natural genetic transformation. To take up extracellular DNA, *S. pneumoniae* assembles competence pili composed of the major pilin ComGC. In addition to ComGC, four minor pilins ComGD, E, F, and G are expressed during bacterial competence, but their role in pilus biogenesis and transformation is unknown. Here, using a combination of protein-protein interaction assays we show that all four proteins can directly interact with each other. Pneumococcal ComGG stabilizes the minor pilin ComGD and ComGF and can interact with and stabilize the major pilin ComGC, thus, deletion of ComGG abolishes competence pilus assembly. We further demonstrate that minor pilins are present in sheared pili fractions and find ComGF to be incorporated along the competence pilus by immunofluorescence and electron microscopy. Finally, mutants of the invariant Glu5 residue (E5), ComGD_E5A_ or ComGE_E5A_, but not ComGF_E5A_, were severely impaired in pilus formation and function. Together, our results suggest that ComGG, lacking E5, is essential for competence pilus assembly and function, and plays a central role in connecting the pneumococcal minor pilins to ComGC.

## Introduction

The transfer of genetic information among bacteria contributes to the natural diversity of prokaryotes and is a major evolutionary driving force. This phenomenon, referred to as horizontal gene transfer (HGT), can occur at high frequency between bacteria that are closely related or that coexist in the same habitat or community (Koonin et al., 2001; Beiko et al., 2005). It allows bacteria to acquire new functional traits that are important in the adaptation and survival of species and plays a role in the spread of antibiotic resistance (Vos et al., 2015; von Wintersdorff et al., 2016; Calero-Caceres et al., 2019). Transformation, one of the main mechanisms of HGT, is a widely conserved process in many bacterial species. The uptake of exogenous DNA from the surrounding environment is entirely controlled by the recipient bacteria and relies on sophisticated DNA uptake machineries often composed of bacterial surface appendages, such as type IV pili (T4P). T4P are widespread among Gram-negative and Gram-positive bacteria and can display a wide range of functions including DNA uptake, adhesion to host cells, biofilm formation and twitching motility (Melville and Craig, 2013; Craig et al., 2019; Pelicic, 2019).

The Gram-positive respiratory pathogen *Streptococcus pneumoniae* (the pneumococcus) is naturally transformable and type IV competence pili promote initial DNA binding during the first step of transformation (Muschiol et al., 2019). These µm-long filaments are exclusively formed during bacterial competence and are essential for DNA uptake. Upon binding of extracellular DNA, the pneumococcal DNA translocation apparatus (transformasome), composed of ComEA, EndA, ComEC and ComFA, facilitates the uptake of DNA across the cellular membrane (Johnston et al., 2014). All the proteins required for competence pilus biogenesis are encoded by the *comG* operon (ComGA, B, C, D, E, F and G) and *pilD* encoded elsewhere in the genome. ComGA is an ATPase, believed to power pilus assembly, and mutants deficient in ComGA are non-transformable (Laurenceau et al., 2013). ComGB is a polytopic integral membrane protein and presumably acts as a platform for pilus assembly based on its homology with known orthologous platform proteins (Takhar et al., 2013). The remaining proteins ComGC, D, E, F and G belong to the family of pilin proteins, a conserved set of proteins found in bacteria expressing T4P and type II secretion systems (T2SSs). Pilins or pseudopilins, as they are referred to in T2SSs, are synthesized as prepilins with a characteristic N-terminal class III signal peptide, which is cleaved off by the prepilin peptidase PilD (Strom et al., 1993; Piepenbrink, 2019). Mature major and minor pilins share a similar domain structure with a conserved N-terminal and a more variable C-terminal domain and are assembled into polymeric filaments by dedicated assembly systems (Giltner et al., 2012; Berry and Pelicic, 2015). The pneumococcal pilins share the typical pilin domain structure but differ in size (86-137 aa). Their signal peptides end with a conserved Ala residue, which is recognized by the prepilin peptidase. All pneumococcal pilins, except ComGG, also contain a conserved Glu residue in position 5 after the cleavage site (E5), which was suggested to be important for fiber assembly/stabilization in other T4P and T2SSs (Strom and Lory, 1991; Aas et al., 2007; Li et al., 2012; Nivaskumar et al., 2016). The pneumococcal competence pilus is composed of the major pilin ComGC. Bacteria deficient in ComGC or expressing mutant ComGC_E5V_ lack pili and are defective in transformation (Laurenceau et al., 2013; Muschiol et al., 2017). The role of the minor pilins in competence pilus biogenesis and transformation remains poorly understood.

Minor pilins are present at lower abundance relative to the major pilin. They can be incorporated into pili at different locations and can have profound effects on pilus assembly and function. Strikingly, many Gram-negative bacteria expressing T4P or T2SSs possess a set of core minor pilins that are central components of the assembly machinery (Giltner et al., 2012; Escobar et al., 2021). In some systems additional accessory minor pilins that can be dispensable for pilus biogenesis can be involved in specific pilus associated functions such as adhesion to host cells, bacterial aggregation, and DNA uptake (Helaine et al., 2005; Helaine et al., 2007; Brissac et al., 2012; Cehovin et al., 2013; Imhaus and Dumenil, 2014; Ng et al., 2016).

Here, we investigate the role of the pneumococcal minor pilins in competence pilus biogenesis and transformation by combining genetic, biochemical, and imaging studies. Our results provide evidence that ComGD, ComGF and ComGG are found in sheared pili fractions, and we demonstrate by immunofluorescence microscopy and electron microscopy that ComGF is incorporated along the pilus filament. We also show that all minor pilins can directly interact with each other and identify ComGG as a link between the minor pilins and the major pilin ComGC.

## Material and Methods

### Bacterial Strains, Growth Conditions and Transformation

All strains used in this study are listed in **Table 1**. *S. pneumoniae* strains were grown on blood agar plates overnight at 37°C and 5% CO_2_. For competence induction, plate-grown bacteria were used to inoculate Todd-Hewitt broth supplemented with 0.5% yeast extract (THY), at OD_620_ = 0.05 and grown at 37°C until OD_620_ = 0.15. Bacteria were then incubated at 30°C for 15 min before competence was induced by addition of competence stimulating peptide (CSP-1) at a final concentration of 100 ng/ml for 15 min. For transformation experiments, DNA (1 μg/ml) was added and transformants were selected on blood agar plates containing appropriate antibiotics, as specified. Transformability assays were performed as previously described (Muschiol et al., 2017).

**Table 1:** *S. pneumoniae* strains used in this study.

Strain	Description	Source/Reference
R6	R6 strain	R. Hakenbeck
R6Δ*comGC*	*comGC*::*spec* (Spec^R^)	This study
R6Δ*comGG*	*comGG*::*spec* (Spec^R^)	This study
R6Δ*comGFG*	*comGFG*::*spec* (Spec^R^)	This study
R6Δ*comGEFG*	*comGEFG*::*spec* (Spec^R^)	This study
R6Δ*comGDEFG*	*comGDEFG*::*spec* (Spec^R^)	This study
T4Δ*comG*	*comG*::*erm* (Erm^R^)	(Balaban et al., 2014)
R6Δ*comG*	*comG*::*erm* (Erm^R^)	This study
R6 *bgaA*::*comGD*	*bgaA*::*P_comG_ comGD* (Tet^R^)	This study
R6 *bgaA*::*comGE*	*bgaA*::*P_comG_comGE* (Tet^R^)	This study
R6 *bgaA*::*comGF*	*bgaA*::*P_comG_comGF* (Tet^R^)	This study
R6 *bgaA*::*comGG*	*bgaA*::*P_comG_comGG* (Tet^R^)	This study
R6 *bgaA*::*comGDEF*	*bgaA*::*P_comG_comGDEF* (Tet^R^)	This study
R6 *bgaA*::*comGDEFG*	*bgaA*::*P_comG_comGDEFG* (Tet^R^)	This study
R6Δ*comGDEFG*, *bgaA*::*comGD*	*comGDEFG*::*spec* (Spec^R^), *bgaA*::*P_comG_ comGD* (Tet^R^)	This study
R6Δ*comGDEFG*, *bgaA*::*comGE*	*comGDEFG*::*spec* (Spec^R^), *bgaA*::*P_comG_comGE* (Tet^R^)	This study
R6Δ*comGDEFG*, *bgaA*::*comGF*	*comGDEFG*::*spec* (Spec^R^), *bgaA*::*P_comG_comGF* (Tet^R^)	This study
R6Δ*comGDEFG*, *bgaA*::*comGG*	*comGDEFG*::*spec* (Spec^R^), *bgaA*::*P_comG_comGG* (Tet^R^)	This study
R6Δ*comGDEFG*, *bgaA*::*comGDEF*	*comGDEFG*::*spec* (Spec^R^), *bgaA::P_comG_comGDEF* (Tet^R^)	This study
R6 *bgaA*::*comGD_Flag_*	*bgaA*::*P_comG_comGD_Flag_* (Tet^R^)	This study
R6 *bgaA*::*comGE_Flag_*	*bgaA*::*P_comG_comGE_Flag_* (Tet^R^)	This study
R6 *bgaA*::*comGF_Flag_*	*bgaA*::*P_comG_comGF_Flag_* (Tet^R^)	This study
R6 *bgaA*::*comGG_Flag_*	*bgaA*::*P_comG_comGG_Flag_* (Tet^R^)	This study
R6Δ*comGG*, *bgaA*::*comGG*	*comGG*::*spec* (Spec^R^)*, bgaA*::*P_comG_comGG* (Tet^R^)	This study
R6 *bgaA*::*comGCDEF*	*bgaA*::*P_comG_comGCDEF* (Tet^R^)	This study
R6 *bgaA*::*comGCDEFG*	*bgaA*::*P_comG_comGCDEFG* (Tet^R^)	This study
R6Δ*comG*, *bgaA*::*comGCDEF*	*comG*::*erm* (Erm^R^), *bgaA*::*comGCDEF* (Tet^R^)	This study
R6Δ*comG*, *bgaA*::*comGCDEFG*	*comG*::*erm* (Erm^R^), *bgaA*::*comGCDEFG* (Tet^R^)	This study
R6 *bgaA*::*comG*	*bgaA*::*comG* (Tet^R^)	This study
R6 *bgaA*::*comG with comGC_E5A_*	*bgaA*::*comG with ComGC_E5A_* (Tet^R^)	This study
R6 *bgaA*::*comG with comGD_E5A_*	*bgaA*::*comG with ComGD_E5A_* (Tet^R^)	This study
R6 *bgaA*::*comG with comGE_E5A_*	*bgaA*::*comG with ComGE_E5A_* (Tet^R^)	This study
R6 *bgaA*::*comG with comGF_E5A_*	*bgaA*::*comG with ComGF_E5A_* (Tet^R^)	This study
R6Δ*comG*, *bgaA*::*comG*	*comG*::*erm* (Erm^R^), *bgaA*::*comG* (Tet^R^)	This study
R6Δ*comG*, *bgaA*::*comG with comGC_E5A_*	*comG*::*erm* (Erm^R^), *bgaA*::*comG with comGC_E5A_* (Tet^R^)	This study
R6Δ*comG*, *bgaA*::*comG with comGD_E5A_*	*comG*::*erm* (Erm^R^), *bgaA*::*comG with comGD_E5A_* (Tet^R^)	This study
R6Δ*comG*, *bgaA*::*comG with comGE_E5A_*	*comG*::*erm* (Erm^R^), *bgaA*::*comG with comGE_E5A_* (Tet^R^)	This study
R6Δ*comG*, *bgaA*::*comG with comGF_E5A_*	*comG*::*erm* (Erm^R^), *bgaA*::*comG with comGF_E5A_* (Tet^R^)	This study

Spec^R^-spectinomycin resistance, Tet^R^-tetracycline resistance, Erm^R^-erythromycin resistance.

*Escherichia coli* strains were grown at 37°C in lysogeny broth (LB) or on LB agar plates containing antibiotics (50 μg/ml kanamycin, 100 μg/ml ampicillin and/or 50 μg/ml chloramphenicol), when required. *E. coli* Top 10 (Life Technologies) was used for cloning with standard techniques as described (Sambrook et al., 1989). For protein expression, *E. coli* T7 Express (NEB) was grown in Terrific Broth (TB) and LB (ratio 1:1) with appropriate antibiotics. Cultures were grown at 37°C to OD_600_ = 0.5, induced with 1 mM isopropyl β-D-1 thiogalactopyranoside (IPTG) purchased from Sigma and continued to grow at room-temperature (RT), overnight (~12 h).

### Generation of Mutants and Complementation Constructs

#### Construction of *S. pneumoniae* R6 Deletion Mutants

Pneumococcal strains were created in which *comGC, comGG*, *comGFG*, *comGEFG* and *comGDEFG* were deleted and replaced with a spectinomycin cassette. For this, PCR fragments flanking the upstream and downstream regions of the target genes were amplified from the *S. pneumoniae* R6 genomic DNA using Fusion Flash polymerase (Thermo Fisher Scientific). The PCR primers contained overhang sequences with BamHI and NotI restriction sites (**Table 2**). Each PCR product was digested with the respective restriction enzyme, purified and ligated to a spectinomycin cassette containing BamHI and NotI sites. The resulting fragment was PCR amplified and used to transform *S. pneumoniae* R6. Transformants were selected on blood agar plates containing spectinomycin (200 µg/ml) and confirmed by PCR and sequencing of the insertion region.

**Table 2:** Primers used in this study.

Name	Sequence	Description
604-F	TTGACTTGCAAGCAGAGATTATCAAG	To construct R6Δ*comGC*
605-R	GCATAGGATCCTTAAAAATTTACCTCCATATTTTGATACATGGGC	To construct R6Δ*comGC*
606-F	CGATTGCGGCCGCGCCTAAGAAAGTTACAAGCAGATGG	To construct R6Δ*comGC*
607-R	GAGGGCAAGCAGGGATTCTAAC	To construct R6Δ*comGC*
372-F	GCATAGGATCCCTAATCAAAATAGTGAGGAGGAGGATATATTTGAATACATAC	To amplify spectinomycin cassette
MSA62-R	CGATTGCGGCCGCTTATAATTTTTTTAATCTGTTATTTAAATAGTTTATAGTTAAATTTACATTTTCATTAG	To amplify spectinomycin cassette
MSA37-F	GCACGCCTTCCCTTTATTGGAATC	To construct R6Δ*comGG*, R6Δ*comGFG*, R6Δ*comGEFG* and R6Δ*comGDEFG*
374-R	GCATAGGATCCCCTGCCTTAACTTTTTTCTTTTTCCACACGATAG	To construct R6Δ*comGG*
361-R	CGATTGCGGCCGCACTAAACGAAATAAACGCTAAAACGTCTC	To amplify spectinomycin cassette
358-F	CGATTGCGGCCGCCTATAATGCGTTGAATCCAGAATAGTCC	To construct R6Δ*comGG*, R6Δ*comGFG*, R6Δ*comGEFG* and R6Δ*comGDEFG*
336-R	CCAAATCACTTTGGATGACTTGGAC	To construct R6Δ*comGG*, R6Δ*comGFG*, R6Δ*comGEFG* and R6Δ*comGDEFG*
368-R	GCATAGGATCCGCCTTAATCATTGACTTTACGATTTGC	To construct R6Δ*comGDEFG*
369-R	GCATAGGATCCCCTAATTTTTTGTTTCCTTAATGCGTTAATTTTTC	To construct R6Δ*comGEFG*
370-R	GCATAGGATCCCCTTATGGCTCTTTGATTGCCAAC	To construct R6Δ*comGFG*
292-F	CCAGCTAAATTGATTATGGTAGACCTAG	To construct R6Δ*comG*
330-R	CCACTTCCATTCCCAAGTAATC	To construct R6Δ*comG*
154-F	CGCCTGCAGGGGTTCTCCTCTACGCAGTCACCATA	To construct pSM159
139-R	CGCGGATCCCTATGAATTCTCTTTCTTTTCAGG	To construct pSM159, pSM200
157-F	CGCCTGCAGGGTTCTCCTCTACGCAGTCACCATA	To construct pSM200
790-F	catcggtacctgcgaattctagAGAAAAGTAACTTTTTTGGAGTTGC	To construct pSM828, pSM850
791-R	cttatcgtcgtcatccttgtaatcATCATTGACTTTACGATTTGCTCC	To construct pSM828
792-F	gcaactccaaaaaagttacttttctCTAGAATTCGCAGGTACCGATG	To construct pSM828, pSM850
793-R	gattacaaggatgacgacgataagCTACCATTACCAGTTGGTCTGG	To construct pSM828
879-F	gcctgttcttccgtgatgcgtccatACTTACCTCCTCACCTATACTATTC	To construct pSM990, pSM992, pSM998
885-R	taaacgcattaaggaaacaaaaaattagCTACCATTACCAGTTGGTCTGG	To construct pSM998
905-R	CTACCATTACCAGTTGGTCTGGTG	To construct pSM974, pSM975, pSM994
882-R	ctatcgtgtggaaaaagaaaaaagttaaCTACCATTACCAGTTGGTCTGG	To construct pSM992
880-F	gaatagtataggtgaggaggtaagtATGGACGCATCACGGAAGAAC	To construct R6 *bgaA*::*comGD* R6 *bgaA*::*comGD_Flag_* R6 *bgaA*::*comGDE* R6 *bgaA*::*comGDEFG*
886-R	aactggtaatggtagCTAATTTTTTGTTTCCTTAATGCGTTTAATTTTTC	To construct R6 *bgaA*::*comGD*
906-F	tagtataggtgaggaggtaagtATGGAAAAATTAAACGCATTAAGGAAAC	To construct R6 *bgaA*::*comGE*, R6 *bgaA*::*comGE_Flag_*
907-R	cagaccaactggtaatggtagTTATGGCTCTTTGATTGCCAACAAC	To construct R6 *bgaA*::*comGE*
908-F	tataggtgaggaggtaagtTTGAGATTCAGGTATTTTCTAGTGAAAAAGG	To construct R6 *bgaA*::*comGF*, R6 *bgaA*::*comGF_Flag_*
883-R	gaccaactggtaatggtagTTAACTTTTTTCTTTTTCCACACGATAGATG	To construct R6 *bgaA*::*comGF*
910-F	agtataggtgaggaggtaagtGTGTGGAAAAAGAAAAAAGTTAAGGCAGG	To construct R6 *bgaA*::*comGG*, R6 *bgaA*::*comGG_Flag_*
911-R	cagaccaactggtaatggtagCTATGAATTCTCTTTCTTTTCAGGCTTC	To construct R6 *bgaA*::*comGG*, R6 *bgaA*::*comGDEFG*
887-F	ttattacttatcgtcgtcatccttgtaatcATTTTTTCCTTAATGCGTTTAATTTTC	To construct R6 *bgaA*::*comGD_Flag_*
912-R	ttattacttatcgtcgtcatccttgtaatcTGGCTCTTTGATTGCCAACAAC	To construct R6 *bgaA*::*comGE_Flag_*
913-R	ttattacttatcgtcgtcatccttgtaatcACTTTTTTCTTTTTCCACACGATAGATGAAC	To construct R6 *bgaA*::*comGF_Flag_*
914-R	ttattacttatcgtcgtcatccttgtaatcTGAATTCTCTTTCTTTTCAGGCTTCTCTTC	To construct R6 *bgaA*::*comGG_Flag_*
904-F	ACTTACCTCCTCACCTATACTATTCGC	To construct pSM974, pSM975, pSM994, R6 *bgaA*::*comGD_Flag,_ E_Flag,_ F_Flag_* or *G_Flag_*
831-R	gattacaaggatgacgacgataagtaataaCTACCATTACCAGTTGGTC	To construct R6 *bgaA*::*comGD_Flag,_ E_Flag,_ F_Flag_* or *G_Flag_*
546-F	GTTAAAGCTTTTACATTGGTGG**C**GATGTTGGTGGTCTTGCTGATTATC	To construct pSM536
547-R	GATAATCAGCAAGACCACCAACATC **G** CCACCAATGTAAAAGCTTTAAC	To construct pSM536
548-F	GGCCTTTACCATGCTGG**C**AAGTCTCTTGGTTTTGGG	To construct pSM545
549-R	CCCAAAACCAAGAGACTT**G**CCAGCATGGTAAAGGCC	To construct pSM545
600-F	GGGCAGTGATTTTACTGG**C**AGCAGTAGTCGCTCTAGCTATC	To construct pSM552
601-R	GATAGCTAGAGCGACTACTGCT**G**CCAGTAAAATCACTGCCC	To construct pSM552
608-F	GTCAAGGCTTTTACCTTGTTAG**C**ATCCCTGCTTGCCCTCATTGTCATCAG	To construct pSM808
609-R	CTGATGACAATGAGGGCAAGCAGGGAT**G**CTAACAAGGTAAAAGCCTTGAC	To construct pSM808
808-R	cagaccaactggtaatggtagCTATGAATTCTCTTTCTTTTCAGGCTTC	To construct pSM850, pSM990,
807-R	gcctgaaaagaaagagaattcatagCTACCATTACCAGTTGGTCTGG	To construct pSM850, pSM990
858-F	GATGACATTCTTGAAAAAAGCTAAGGTTAAAGC	To construct pSM915, pSM913
859-R	CTACTGCTTCCAGTAAAATCACTGCC	To construct pSM915, pSM913
856-F	GGCAGTGATTTTACTGGAAGCAGTAG	To construct pSM915, pSM913
857-R	GCTTTAACCTTAGCTTTTTTCAAGAATGTCATC	To construct pSM915, pSM913
845-F	ATGGACGCATCACGGAAGAAC	To construct pSM872
846-R	CGTAGAGGAGAACACCTGCC	To construct pSM872, pSM903
843-F	GGCAGGTGTTCTCCTCTACG	To construct pSM872, pSM903
844-R	GTTCTTCCGTGATGCGTCCAT	To construct pSM872
341-F	GAAAAAGGATTGGAGGTCTACCATGG	To construct pSM903
860-R	CCATGGTAGACCTCCAATCCTTTTTC	To construct pSM903
413-F	CGCGGATCCGAGTCAGCTCCTCATTTCAGAAGTTC	To construct pSM507
414-R	CGCAAGCTTTTAACTTTTTTCTTTTTCCACACGATAGATGAAC	To construct pSM507
415-F	CGCGGATCCGTTGAACCGACAAGTCGCCC	To construct pSM429
416-R	CGCAAGCTTCTATGAATTCTCTTTCTTTTCAGGCTTCTCTTC	To construct pSM429
417-F	CGCCATATGGTAGAGGAACAGATTTTCTTTATGGAGTTTGAAG	To construct pSM507
418-R	CGCCTCGAGCTAATTTTTTGTTTCCTTAATGCGTTTAATTTTTCCATTTC	To construct pSM507
419-F	CGCCATATGCAAAAAAATAGGCAAGAGGAAGCAAAAATC	To construct pSM429
420-R	CGCCTCGAGTTATGGCTCTTTGATTGCCAACAACTG	To construct pSM429
49-F	CGCGGATCCACCAAGCAAAAAGAAGCAGTCA	To construct pSM29
2-R	CGCCTCGAGTTAATCATTGACTTTACGATTTGC	To construct pSM29
50-F	CGCGGATCCGGCTCTGTCCAGTCCACTTTTT	To construct pSM34
4-R	CGCCTCGAGCTAATTTTTTGTTTCCTTAATGCG	To construct pSM34, pMB13
51-F	CGCGGATCCCAAATTCAAAAAAATAGGCAAG	To construct pSM51
6-R	CGCCTCGAGTTATGGCTCTTTGATTGCCAACAA	To construct pSM51, pMB17
52-F	CGCGGATCCCAAGCTATGAGTCAGCTCCTCA	To construct pSM40
8-R	CGCCTCGAGTTAACTTTTTTCTTTTTCCACACG	To construct pSM40, pMB21
53-F	CGCGGATCCTTGAACCGACAAGTCGCCCACT	To construct pSM46
10-R	CGCCTCGAGCTATGAATTCTCTTTCTTTTCAGG	To construct pSM46, pMB22
14-F	CGCCATATGGGCTCTGTCCAGTCCACTTTTT	To construct pMB13
15-F	CGCCATATGCAAATTCAAAAAAATAGGCAAG	To construct pMB17
16-F	CGCCATATGCAAGCTATGAGTCAGCTCCTCA	To construct pMB21
17-F	CGCCATATGTTGAACCGACAAGTCGCCCACT	To construct pMB22
258-F	ATTAGACCGTTCGCAGTTC	To assess *ComGF* expression
259-R	CGAACCAGTTGATTGTCCT	To assess *ComGF* expression
gryA-F	GTTCGCTTGGTTCAGGAAAA	To assess *gyrA* expression
gryA-R	TTGCATTTGGGTCATTTTGA	To assess *gyrA* expression

Overhangs are shown in lower case. Mismatched bases generating mutations are in bold.

Mutant strains lacking the entire *comG* operon were produced by transformation with a PCR product containing *comG* up and downstream flanking regions fused to an erythromycin cassette amplified from T4Δ*comG* (Balaban et al., 2014) genomic DNA with primers 292-F/330-R. Clones were selected on blood agar plates containing erythromycin (1 µg/ml) and confirmed by sequencing.

#### Construction of Δ*comGDEFG* Mutant Strains Ectopically Expressing Minor Pilin(s) in the *bgaA* Locus

PCR and *in vivo* recombination in *E. coli* were used to generate integration vectors carrying pneumococcal minor pilin(s) for subsequent transformation into the *S. pneumoniae* strain R6. Based on the integration vector pJVW25 [kindly provided by A. Eberhardt (Eberhardt et al., 2009)], we first created a modified plasmid containing the flanking regions for integration into the *bgaA* locus but replacing the Zn^2+^ inducible promoter and *gfp* with the sequence encoding the *comG* promoter, *comGA*, *comGB* and *comGC_Flag_*. The respective fragment of the pJVW25 plasmid was amplified with Fusion Flash polymerase (Thermo Fisher Scientific) and primers 792-F/793-R that contained overhang sequences homologous to the *comG* promoter and the Flag tag, respectively. The insert was amplified from the *S. pneumoniae* R6 genomic DNA with primers 790-F/791-F carrying overhang sequences homologous to the pJVW25 plasmid and the Flag-tag. Insert and PCR amplified plasmid were then mixed in a 1:1 molar ratio and treated with *Dpn*I for 1 h at 37°C. 1µl of the reaction was used to transform XL10-gold competent cells (Stratagene) that combine vector and insert by homologous recombination. Clones were selected on LB plates containing ampicillin (100 µg/ml) and the plasmid was isolated using the QIAprep Spin Miniprep Kit (Qiagen) according to the manufacturer’s instructions. The resulting plasmid was designated pSM828 and used as template to produce integration vectors carrying minor pilin(s) as follows: *comGD*, *comGE*, *comGF*, *comGG*, *comGDEF* and *comGDEFG*, which were PCR amplified from R6 genomic DNA using the following primers: 880-F/886-R, 906-F/907-R, 908-R/883-R, 910-F/911-R, 880-F/883-R and 880-F/911-R. The respective plasmid fragments were amplified from pSM828 using primers 879-F/885-R (*bgaA*::*comGD*), 904-F/905-R (*bgaA*::*comGE, bgaA*::*comGF* and *bgaA*::*comGG*), 879-F/882-R (*bgaA*::*comGDEF*) and 879-F/807-R (*bgaA*::*comGDEFG*) (**Table 2**). PCR amplified inserts and vectors were combined as described above. The resulting integration vectors, pSM998, pSM994, pSM975, pSM974, pSM992 and pSM990 (**Table 3**) were used for transformation of *S. pneumoniae* R6. Transformants were selected on blood agar plates containing tetracycline (2 µg/ml). Finally, endogenous *comGDEFG* was deleted in the obtained strains (**Table 1**) as described earlier for the R6Δ*comGDEFG* mutant. The resulting strains lacking endogenous *comGDEFG* and expressing minor pilin(s) in the *bgaA* locus are listed in **Table 1**. All plasmids and strains were confirmed by PCR and DNA sequencing.

**Table 3:** Plasmids used in this study.

Name	Description	Source
pJVW25	Integration vector	(Eberhardt et al., 2009)
pSM828	Integration vector encoding *comG* promoter (P*_comG_*), *comGA*, *comGB* and *comGC_Flag_*	This study
pSM998	Integration vector encoding *P_comG_ comGD*	This study
pSM994	Integration vector encoding *P_comG_ comGE*	This study
pSM975	Integration vector encoding *P_comG_ comGF*	This study
pSM974	Integration vector encoding *P_comG_ comGG*	This study
pSM992	Integration vector encoding *P_comG_ comGDEF*	This study
pSM990	Integration vector encoding *P_comG_ comGDEFG*	This study
pSM1021	Integration vector encoding *P_comG_ comGD_Flag_*	This study
pSM1027	Integration vector encoding *P_comG_ comGE_Flag_*	This study
pSM976	Integration vector encoding *P_comG_ comGF_Flag_*	This study
pSM971	Integration vector encoding *P_comG_ comGG_Flag_*	This study
pSM850	Integration vector encoding *comG* operon	This study
pSM526	pCR-Blunt-II-Topo encoding *comGCDEFG*	This study
pSM536	pCR-Blunt-II-Topo encoding *comGC_E5A_DEFG*	This study
pSM545	pCR-Blunt-II-Topo encoding *comGCD_E5A_EFG*	This study
pSM552	pCR-Blunt-II-Topo encoding *comGCDE_E5A_FG*	This study
pSM808	pCR-Blunt-II-Topo encoding *comGCDEF_E5A_G*	This study
pSM915	Integration vector encoding *comG* operon with *comGC_E5A_*	This study
pSM913	Integration vector encoding *comG* operon with *comGD_E5A_*	This study
pSM872	Integration vector encoding *comG* operon with *comGE_E5A_*	This study
pSM903	Integration vector encoding *comG* operon with *comGF_E5A_*	This study
pSM429	pACYCDuet-1 expressing ComGE_25-86_ and 6x-His ComGG_21-128_	This study
pSM507	pETDuet expressing comGD_31-130_ and 6x-His comGF_24-137_	This study
pSM29	pGEX4T1 expressing GST-ComGC_25-93_	This study
pSM34	pGEX4T1 expressing GST-ComGD_22-130_	This study
pSM51	pGEX4T1 expressing GST-ComGE_23-86_	This study
pSM40	pGEX4T1 expressing GST-ComGF_21-137_	This study
pSM46	pGEX4T1 expressing GST-ComGG_21-128_	This study
pMB13	pET21a expressing ComGD_22-130_	This study
pMB17	pET21a expressing ComGE_23-86_	This study
pMB21	pET21a expressing ComGF_21-137_	This study
pMB22	pET21a expressing ComGG_21-128_	This study
pKT25	BACTH vector designed to express a protein fused to the C-terminal end of T25	(Karimova et al., 1998)
pUT18C	BACTH vector designed to express a protein fused to the C-terminal end of T18	(Karimova et al., 1998)
pKT25-zip	Vector expressing the leucine zipper of GCN4 fused to T25 (control plasmid)	(Karimova et al., 1998)
pUT18C-zip	Vector expressing the leucine zipper of GCN4 fused to T18 (control plasmid)	(Karimova et al., 1998)
pKT25-*comGC*	Vector expressing T25-ComGC	(Muschiol et al., 2017)
pUT18C-*comGC*	Vector expressing T18-ComGC	(Muschiol et al., 2017)
pSM159	pKT25 expressing T25-ComGG_2-128_	This study
pSM200	pUT18C expressing T18-ComGG_2-128_	This study

#### Construction of Strains Ectopically Expressing Flag-Tagged Minor Pilin(s) in the *bgaA* Locus

Using the same cloning strategy as described above, we generated strains that expressed Flag-tagged minor pilins in the *bgaA* locus. In brief, *comGD*, *comGE*, *comGF* and *comGG* were amplified from R6 genomic DNA with Fusion Flash polymerase (Thermo Fisher Scientific) and the following primers: 880-F/887-R, 906-F/912-R, 908-F/913-R and 910-F/914-R (**Table 2**). The sequence encoding the Flag tag was added to the reverse primers. The vector was amplified from pSM828 using primers: 904-F/831-R (**Table 2**). The PCR amplicons for the inserts were combined with their respective PCR amplified vector and resulting plasmids pSM1021, pSM1027, pSM976 and pSM971 (**Table 3**) were used for transformation of *S. pneumoniae* R6. Transformants were selected on blood agar plates containing tetracycline (2 µg/ml) and confirmed by PCR and sequencing.

#### Construction of the R6Δ*comGG* Complemented Strain Expressing *comGG* in the *bgaA* Locus

The complemented R6Δ*comGG*, *bgaA*::*comGG* strain was produced in the R6 *bgaA*::*comGG* strain background by deletion mutagenesis as described for the construction of *S. pneumoniae* deletion mutants. Transformants were confirmed by PCR and sequencing.

#### Construction of Δ*comG* Strains Ectopically Expressing the *comG* Operon and E5A Minor Pilin Variants in the *bgaA* Locus

First, an integration vector was constructed that would allow integration of the entire *comG* operon into the *bgaA* locus of the *S. pneumoniae* strain R6. For that, the *comG* operon was PCR amplified with primers 790-F/808-R from genomic R6 DNA and the vector was amplified from pJVW25 with primers 792-F/807-R. Both fragments were combined as described earlier. The resulting plasmid (pSM850) was transformed into *S. pneumoniae* R6 to create R6 *bgaA*::*comG* and then the endogenous *comG* operon was deleted essentially as described above. R6Δ*comG*, *bgaA*::*comG* variants carrying a E5A point mutation in either *comGC*, *comGD*, *comGE* or *comGF* were constructed following the same approach. To introduce the point mutations, a plasmid encoding *comGC* to *comGG* (pSM526) was used as template for site-directed mutagenesis with primers: 546-F/547-R (*comGC_E5A_*), 548-F/549-R (*comGD_E5A_*), 600-F/601-R (*comGE_E5A_*) and 608-F/609-R (*comGF_E5A_*). The resulting plasmids (pSM536, pSM545, pSM552 and pSM808) were then used to create integration vectors encoding *comG* with *comGC_E5A_*, *comGD_E5A_*, *comGE_E5A_* or *comGF_E5A_*, respectively. Insert and vector backbone were PCR-amplified as follows: for *comGC_E5A_* (insert: primers 858-F/859-R on pSM536, vector: primers 856-F/857-R on pSM850), *comGD_E5A_* (insert: primers 858-F/859-R on pSM545, vector: primers 856-F/857-R on pSM850), *comGE_E5A_* (insert: primers 845-F/846-R on pSM552, vector: primers 843-F/844-R on pSM850) and *comGF_E5A_* (insert: primers 341-F/846-R on pSM808, vector: primers 843-F/860-R on pSM850) and combined as described above. The resulting integration vectors pSM915, pSM913, pSM872 and pSM903 (**Table 3**) were then used for transformation of *S. pneumoniae* R6 to create R6 *bgaA*::*comG with comGC_E5A,_* R6 *bgaA*::*comG with comGD_E5A,_* R6 *bgaA*::*comG with comGE_E5A,_* and R6 *bgaA*::*comG with comGF_E5A_* (**Table 1**). In the last step, the endogenous *comG* operon was removed in these strains as described above, producing R6Δ*comG*, *bgaA*::*comG with comGC_E5A_*, R6Δ*comG*, *bgaA*::*comG with comGD_E5A_*, R6Δ*comG*, *bgaA*::*comG with comGE_E5A_* and R6Δ*comG*, *bgaA*::*comG with comGF_E5A_* (**Table 1**).

#### Construction of Co-Expression Plasmids for Co-Immunoprecipitation

For co-expression of the soluble domains of ComGD, ComGE, ComGF and ComGG in *E. coli*, the pACYCDuet-1 (Novagen) and the pETDuet-1 vector (Novagen) were used and plasmids were constructed as follows. The respective minor pilins were amplified by PCR from T4 genomic DNA with Fusion Flash polymerase (Thermo Fisher Scientific) and primers 417-F/418-R (*comGD*), 419-F/420-R (*comGE*), 413-F/414-R (*comGF*) and 415-F/416-R (*comGG*) (**Table 2**). The cloning is based on the T4 genome, because *comGE* is not annotated in the *S. pneumoniae* R6 strain. All PCR primer pairs contained overhang sequences with NdeI/XhoI or BamHI/HindIII restriction sites (**Table 2**). PCR products and plasmids were then digested with respective restriction enzymes and the target genes were sequentially inserted into pACYCDuet-1 and pETDuet-1 by ligation. The resulting plasmids, pSM429 encoding the soluble domain of ComGE and 6x-His tagged ComGG and pSM507 encoding the soluble domain of ComGD and 6x-His tagged ComGF (**Table 3**) were co-transformed into competent *E. coli* T7 Express for subsequent protein expression and co-immunoprecipitation.

#### Construction of Plasmids for Pull-Down Studies in *E. coli*

To express the soluble domain of each pilin as GST-tagged fusion or untagged protein in *E. coli.*, the corresponding sequence of each pilin gene was amplified by PCR from TIGR4 (T4) genomic DNA with Fusion Flash polymerase (Thermo Fisher Scientific) and primers listed in **Table 2**. All PCR primer pairs contain overhang sequences with BamHI/XhoI or NdeI/XhoI restriction sites. PCR products were digested with NdeI/XhoI for cloning into pGEX4T1 or BamHI/XhoI for cloning into pet21a. Vectors were cut with respective enzymes. The resulting recombinant plasmids (**Table 3**) were confirmed by sequencing and transformed into competent *E. coli* T7 Express for protein expression.

#### Plasmid Construction for Bacterial Two Hybrid (BACTH) Assays

Vectors expressing T25-ComGC, T18-ComGC were previously described (Muschiol et al., 2017). The gene encoding mature ComGG was PCR-amplified from *S. pneumoniae* genomic DNA using primers 154-F/139-R and 157-F/139-R (**Table 2**). PCR products were digested with PstI/BamHI and cloned into the same sites of pKT25 and pUT18C vector. All strains used for BACTH are listed in **Table 3**.

### Preparation of Cell Extracts and Sheared Pili

Bacteria were grown until OD_620_ = 0.15 and competence was induced as described above. Whole cell lysates were prepared from 10 ml cultures if not stated otherwise. Competent bacteria were harvested by centrifugation for 10 min at 2,700 *g* and 4°C. Bacterial pellets were resuspended in 100 µl of Lysis buffer containing 50 mM Tris pH 8.0 and 3% sodium dodecyl sulphate (SDS). Protein concentration was determined using a NanoDrop ND 1000 (Thermo Fisher Scientific). Samples containing 10 µg/µl total protein were resuspended in 4x NuPAGE^®^ LDS Sample Buffer (Life Technologies) and used for SDS-PAGE and immunoblotting. Sheared pili were prepared from a 250 ml culture of competent bacteria. Pellet and supernatant were separated by centrifugation for 30 min at 4,000 *g* at 4°C. The supernatant was removed. The pellet was resuspended in 1 ml PBS containing cOmplete™ protease inhibitor cocktail (Roche) and sheared by pipetting (1 min) to release pili into the medium. This suspension was centrifuged once at 16,000 *g* for 10 min and the pellet was discarded. The sheared pili fraction was further centrifuged twice at 16,000 *g* for 5 min to remove residual bacteria.

### Antibodies

Antisera against pneumococcal ComGC and ComGG has previously been described (Balaban et al., 2014). Anti-GAPDH rabbit polyclonal antibody was generously provided by P. Mellroth, MTC, Karolinska Institutet. The monoclonal anti-Flag M2 antibody was purchased from Sigma. Polyclonal anti-ComGD and anti-ComGF antiserum was produced by immunizing rabbits with a synthetic peptide, CGNGKIKRIKETKN (anti-ComGD) and CGKSSDDFRKTNAR-NH2 (anti-ComGF) (Innovagen AB). Polyclonal anti-ComGE peptide antiserum was produced against the synthetic peptide KNRQEEAKILQKEEC and affinity purified (Genscript).

### SDS-PAGE and Immunoblotting

Proteins were separated in NuPAGE™ 4-12% Bis-Tris Protein Gels (Thermo Fisher Scientific) and transferred onto PVDF membranes (Trans-Blot^®^ Turbo™ Midi PVDF Transfer Packs, Bio-Rad) using semi-dry electrotransfer (Bio-Rad). Membranes were blocked with 5% milk in PBS containing 0.1% Tween-20 (PBST) and incubated with specific antiserum (anti-Flag 1/2000, anti-ComGC 1/2000, anti-ComGD 1/1000, anti-ComGE 1/1000, anti-ComGF 1/1000, anti-ComGG 1/1000 and anti-GAPDH 1/2000) followed by incubation with respective secondary antibodies diluted in PBST. The HRP-conjugated secondary anti-rabbit and anti-mouse antibody (GE Healthcare) and the HRP-conjugated secondary anti-goat antibody (Sigma) were used at a 1/10 000 dilution. Membranes were developed with ECL Plus™ Western Blotting Reagent (GE Healthcare) and chemiluminescence was recorded using a ChemiDoc™ XRS+ Gel Documentation System (Bio-Rad).

### BACTH Assay

Plasmids encoding fusions to T25 and T18 (**Table 3**) were co-transformed into competent *E. coli* BTH101 (Euromedex) and selected on LB plates containing 50 μg/ml kanamycin and 100 μg/ml ampicillin. Transformants were used for BACTH assays and the functional complementation between the recombinant plasmids was determined by measuring β-galactosidase activity in liquid cultures as described previously (Muschiol et al., 2017).

### RNA Extraction and Real-Time Quantitative PCR (qPCR) Analysis

*S. pneumoniae* strains were grown in 10 ml THY cultures, and competence was induced as described. RNA extraction was performed using the FastRNA Pro™ Blue Kit (MPBio) according to the manufacturer’s instructions. After extraction, the concentration and purity of isolated RNA was determined, and 10 μg of RNA from each sample was DNAse treated using the Turbo DNA-free™ Kit (Thermo Scientific). cDNA was synthesized using 1 μg of RNA and the High-Capacity cDNA Reverse Transcription Kit according to the manufacturer’s protocol (Applied Biosystems™). qPCR was performed using the iTaq™ Universal SYBR^®^ Green Supermix (Bio-Rad). Expression levels of *comGF* were assessed with primers 258-F/259-R. The specificity of each primer pair was validated by melt curve analysis of the PCR product to guarantee the absence of primer dimers and unspecific products. The mRNA expression levels were normalized to the levels of *gyrA* and the relative expression determined with the ΔΔCT method. A total of three biological replicates were included for each strain tested.

### Pull-Down of GST-Tagged Proteins

All *E. coli* strains and plasmids used in this experiment are listed in **Table 1** and **Table 3**, respectively. Protein expression was performed as described above (see, *Bacterial strains, growth conditions and transformation*). Bacteria were harvested by centrifugation and pellets were stored at -20°C. Pellets corresponding to similar amounts of protein, depending on the efficiency of protein expression, were mixed. In this way, approximately equal amounts of expressed protein were combined. Pellets were resuspended in 15 ml GST-buffer containing PBS and cOmplete™ protease inhibitor cocktail (Roche) and the pellet from a culture expressing an untagged pilin was mixed with the pellet expressing a GST-tagged pilin. Combined pellets were lysed using a Stansted cell disrupter (Homogenising Systems Limited) and spun at 20,000 *g* for 30 min at 4°C. Empty gravity flow columns (Bio-Rad) were filled with 0.5 ml Glutathione Sepharose™ 4 Fast Flow resin (GE Healthcare) and equilibrated with GST-buffer. Bacterial supernatant was passed over the column, followed by a washing step with 15 ml GST-buffer. Elution was performed with 0.7 ml of GST-buffer containing 10 mM L-glutathione (Sigma). Samples were analyzed by SDS-PAGE and Coomassie brilliant blue staining and immunoblotting for ComGD, ComGE and ComGG.

### Immunoprecipitations

#### Co-Immunoprecipitation of Pneumococcal Sheared Pili

Protein A Sepharose^®^ 4 Fast Flow (GE Healthcare) was washed three times with PBS buffer and blocked with PBS containing 5% bovine serum albumin (BSA) for 1 h at 4°C. Anti-ComGC antibodies were then bound to Protein A Sepharose, rotating for 1 h at 4°C. ComGC-coupled protein A resin was washed three times with PBS and incubated with sheared pili (200 µl, see Preparation of cell extracts and sheared pili) from *S. pneumoniae* R6 or *S. pneumoniae* R6Δ*comGC* (negative control) on a rotating wheel overnight at 4°C. After five washing steps with PBS containing cOmplete™ protease inhibitor cocktail (Roche), the resin was directly resuspended in 1x NuPAGE^®^ LDS Sample Buffer (Life Technologies) and boiled for 10 min. Samples were analyzed by SDS-PAGE and immunoblotting for ComGC, ComGD, ComGF and ComGG.

#### Co-Immunoprecipitation of Minor Pilins Recombinantly Expressed in *E. coli*

*E. coli* T7 Express competent cells were co-transformed with pSM429 and pSM507 and clones were selected on LB plates containing 100 μg/ml ampicillin and 50 μg/ml chloramphenicol. Positive clones were used for protein expression as described above. A pellet from a 250 ml culture was resuspended in 30 ml PBS containing cOmplete™ protease inhibitor cocktail (Roche) and lysed using a Stansted cell disrupter (Homogenising Systems Limited). Pellet (insoluble fraction) and supernatant (soluble fraction) were separated by centrifugation at 20,000 *g* for 30 min at 4°C. The supernatant was filtered and incubated with Dynabeads™ Protein G (Thermo Fisher Scientific) coupled with ComGG antibodies for 1h at 4°C. Supernatant incubated with uncoupled Dynabeads™ Protein G was used as a negative control. After three washing steps with PBS containing cOmplete™ protease inhibitor cocktail (Roche), the resin was directly resuspended in 1x NuPAGE^®^ LDS Sample Buffer (Life Technologies) and boiled for 10 min. Samples were analyzed by SDS-PAGE and Coomassie brilliant blue staining and immunoblotting for ComGD, ComGE, ComGF and ComGG.

### Immunofluorescence Microscopy

Bacteria were grown until OD_620_ = 0.15 and competence was induced as described above. 10 ml of culture were harvested by centrifugation for 5 min at 2,700 *g*, 4°C. The pellet was resuspended in 5 ml PBS containing 0.5% BSA. 10 µl of this suspension was placed on a coverslip (VWR) and air-dried. Bacteria were fixed in PBS containing 4% paraformaldehyde for 30 min and coverslips were washed three times with PBS containing 0.5% BSA. Samples were labeled with primary antibodies to ComGC, ComGD, ComGF or ComGG, diluted at 1:300, and secondary goat anti-rabbit antibody conjugated with Alexa Fluor 488 or Alexa Fluor 594 (Life technologies) or secondary rabbit anti-goat antibody conjugated with Alexa Fluor 488 (Abcam) for 1 h. Flag-tagged proteins were labeled with anti-Flag M2 monoclonal antibody (Sigma) diluted 1:300 and secondary goat anti-mouse antibody conjugated with Alexa Fluor 594 for 1h. Coverslips were mounted onto microscope slides with Vectashield (Vector Laboratories). Samples were examined using a DV Elite microscope (Applied Precision) equipped with a scientific complementary metal-oxide-semiconductor (sCMOS) camera. Images were acquired using Softworx (Applied Precision) and analyzed in ImageJ (Schneider et al., 2012).

### Transmission Electron Microscopy and Immunogold Labeling

Negative-stain electron microscopy and immunogold labeling was performed as previously described (Muschiol et al., 2017). For immunogold labelling anti-ComGF antibodies, diluted 1:100 and secondary goat anti-rabbit antibodies conjugated to 6-nm gold particles were used. Images were acquired using a Tecnai 12 Spirit Bio TWIN transmission electron microscope (FEI Company, Eindhoven, Netherlands) operated at 100 kV and a Veleta 2k x 2k camera (Olympus Soft Imaging Solutions, GmbH, Münster, Germany).

### Statistical Analysis

Results are expressed, as mean ± SD. Data were statistically analyzed using GraphPad Prism 5.04 software. Statistical analysis was done by one-way ANOVA with subsequent Dunnett’s *post hoc* test (BACTH assays) or two-tailed Student’s t-test (transformability assays) as indicated in the figure legends. Asterisks in the figures indicate groups of statistically different means (* *p ≤* 0.05, ** *p ≤* 0.01, *** *p ≤* 0.001).

## Results

### Lack of ComGG Blocks Pilus Formation and Transformation

**Figure 1:**
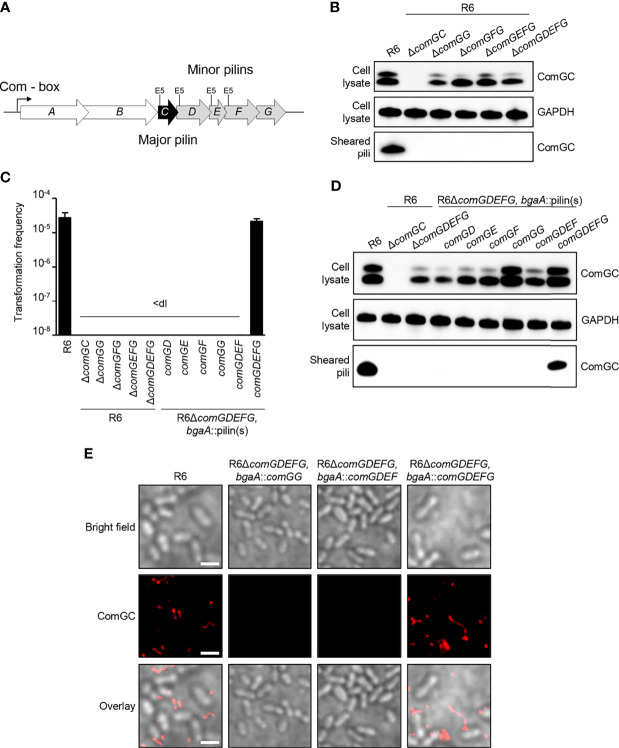
Competence pilus assembly and transformability depends on the pneumococcal minor pilins. **(A)** Schematic representation of the *comG* operon in *S. pneumoniae* R6 strain encoding *comGA* and *comGB* (white), the major pilin gene *comGC* (black) and the minor pilin genes *comGD, comGE, comGF* and *comGG* (grey). The invariant Glu5 residue, present in all pneumococcal pilins except ComGG, is highlighted (E5). **(B)** ComGC was detected by immunoblotting in bacterial whole cell lysates and sheared pili of mutants lacking Δ*comGC*, Δ*comGG*, Δ*comGFG*, Δ*comGEFG*. The WT strain was included as a positive control and GAPDH as loading control. **(C)** Transformation frequency of WT*, comG* mutants and R6Δ*comGDEFG* complemented strains. The detection limit (dl) of the assay was 2.39e^-9^ and the error bars represent the standard deviation of a minimum of three independent experiments (n=3). **(D)** Western blotting analysis of ComGC in bacterial whole cell lysates and sheared pili of Δ*comGDEFG* strains expressing individual minor pilins, *comGDEF* or *comGDEFG* at the ectopic *bgaA* locus. The WT strain was included as a positive control and GAPDH as loading control. **(E)** Immunofluorescence microscopy (IF) of competence-induced pili in WT R6 and R6Δ*comGDEFG* complemented with *comGG, comGDEF or comGDEFG.* Bacteria were visualized by bright field (BF) microscopy and competence pili were labelled with antisera specific for ComGC (red). Scale bars represent 2 µm.

The *S. pneumoniae* minor pilins are encoded in the CSP (competence stimulating peptide)-inducible polycistronic *comG* operon. It consists of seven genes, encoding the ATPase ComGA, and the membrane protein ComGB, the major pilin ComGC and the four minor pilins, ComGD, ComGE, ComGF and ComGG (**Figure 1A**). Since all pilin genes are overlapping, we constructed a series of minor pilin deletion strains by truncating the 3´ end of the comG operon and tested their effect on pilus formation and transformability. As shown in **Figure 1B**, deletion of *comGG* led to overall reduced levels of ComGC present in bacterial whole-cell lysates, while ComGC processing was not visibly affected. The upper band corresponds to unprocessed and the lower band to processed ComGC. No ComGC was found in the sheared pili fraction of the minor pilin mutants and the control strain deficient in ComGC (R6*ΔcomGC*), suggesting that competence pili are not produced in these strains. Consistent with this observation, all strains were non-transformable compared to the WT R6 strain (**Figure 1C**). To test whether pilus assembly and transformation can be restored in a non-transformable *ΔcomGDEFG* strain, either single minor pilins, ComGDEF or ComGDEFG, were expressed from their native CSP-inducible promoter at the ectopic *bgaA* locus. The relative abundance of ComGC was assessed in whole-cell lysates and sheared pili fractions by immunoblotting. As shown in **Figure 1D**, ComGC was detected in lower amounts in bacterial lysates of strains expressing single *comGD*, *comGE* or *comGF*, and in the strain expressing *comGDEF* compared to the WT R6 strain, suggesting that ComGC is not stable in these strains. In the complementation strain ectopically expressing *comGG* alone or *comGDEFG*, similar levels of ComGC were detected in the lysate compared to the WT strain, which indicates a stabilizing effect of ComGG on ComGC. We then assessed the presence of ComGC in the sheared pili fraction of these strains and detected ComGC only in the complemented *comGDEFG* strain (**Figure 1D**), suggesting that all four minor pilins are required to restore piliation. Consistent with this result, the *comGDEFG* complemented strain was transformable to WT levels (**Figure 1C**), and competence pili were detectable by immunofluorescence microscopy (**Figure 1E**).

### ComGG Can Interact With ComGC and Is Required for Stabilization of ComGD and ComGF

**Figure 2:**
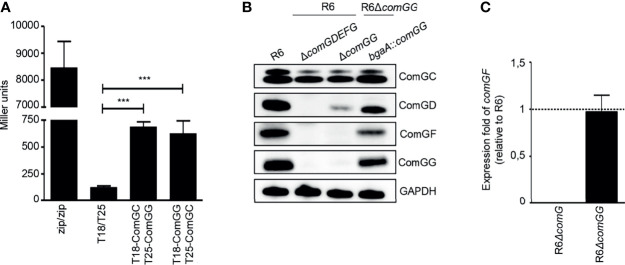
ComGG interacts with ComGC and is required for the stability of ComGD and ComGF. **(A)** Quantification of the interaction between ComGG and ComGC by BACTH. The graph shows mean values of β-galactosidase activity expressed in Miller units for the hybrid proteins T18-ComGC/T25-ComGG and T18-ComGG/T25-ComGC. A strain expressing Zip-T18 and Zip-T25 (zip/zip), in which the hybrid proteins interact through a leucine zipper motif, was included as a positive control. *E. coli* BTH101 co-transformed with pUT18C and pKT25 empty plasmids was used as a negative control. The error bars represent the standard deviation of three independent experiments (n=3) with three different clones. A one-way analysis of variance test followed by Dunnett’s post-test to compare each interaction pair to the negative control (T18/T25) was used for statistical analysis: ***p ≤ 0.001. **(B)** ComGC, ComGD, ComGF and ComGG were detected by immunoblotting in bacterial whole cell lysates in the WT R6 strain, a mutant lacking all minor pilins (*ΔcomGDEFG*) or *comGG*, and a complemented Δ*comGG* mutant strain that ectopically expressed *comGG* from the *bgaA* locus (R6Δ*comGG, bgaA*::*comGG*). GAPDH was used as loading control. **(C)** Relative expression of *comGF* in competent R6*ΔcomG* and R6*ΔcomGG.* The R6*ΔcomG* operon mutant strain was included as negative control. Data was normalized to *gyrA* using the 2^-ΔΔ^ *^CT^* method and presented as the mean fold change of *comGF* relative to the WT R6 strain. Error bars represent the standard deviation of three independent experiments (n=3). The dotted line indicates an expression ratio of 1.

Because ComGG alone was able to stabilize the major pilin ComGC in a strain lacking the endogenous minor pilins (R6*ΔcomGDEFG, bgaA*::*comGG)*, we reasoned that ComGG and ComGC likely interact directly. To test this hypothesis, we used a bacterial adenylate cyclase two-hybrid assay (Karimova et al., 1998) in which the T18 and T25 fragments of *Bordetella pertussis* adenylate cyclase (CyaA) were fused to the N-terminal end of mature ComGC and ComGG. Compared to the negative control (T25/T18), T25-ComGC/T18-ComGG and T18-ComGC/T25-ComGG showed a statistically significant increase in CyaA activity (**Figure 2A**), suggesting a direct interaction between these two pilins. We also tested T18 and T25 fusions for the remaining minor pilins, but these constructs were not expressed in BTH101 *E. coli* cells.

Despite its importance, ComGG alone was not sufficient to restore pilus assembly in a *ΔcomGDEFG* mutant and only complementation with all four minor pilins could rescue the defect. We therefore reasoned that if ComGG and the other minor pilins interact directly, then their stability might be mutually dependent. To test this idea, we studied the stability of the other minor pilins in the *ΔcomGG* deletion mutant using antisera against ComGD, ComGE, ComGF and ComGG. To verify the specificity of the antisera a strain lacking all four minor pilins was included. As can be seen in **Figure 2B**, the respective antisera detected ComGD, ComGF and ComGG in the WT strain, but not in the *ΔcomGDEFG* mutant. ComGE antisera failed to detect specific protein in pneumococcal lysates but recognized recombinant ComGE. Surprisingly, in the *ΔcomGG* deletion mutant the levels of ComGD were strongly reduced and ComGF was entirely absent. Since we were able to detect the *comGF* transcript by qPCR in this strain (**Figure 2C**), the observed effect suggests that ComGG is required for the stability of the other minor pilins. In the complemented mutant with ComGG ectopically expressed under the transcriptional control of the native CSP-inducible promoter, expression of ComGD and ComGF was restored, albeit not at WT levels (**Figure 2B**). These results show that deletion of ComGG has a negative impact on the stability of ComGD and ComGF, supporting evidence that the minor pilins directly interact.

### Pneumococcal Minor Pilins Interact Directly

**Figure 3:**
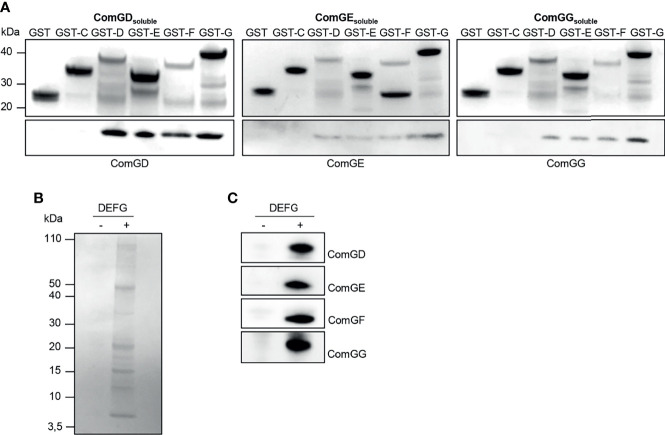
Soluble domains of pneumococcal minor pilins interact directly. **(A)** Untagged (ComGD_soluble_, ComGE_soluble_ or ComGG_soluble_) or GST-tagged C-terminal, soluble domains of the pneumococcal pilins (GST-C, GST-D, GST-E, GST-F or GST-G) were expressed individually in *E. coli.* Pellets expressing untagged protein were mixed with pellets expressing GST-tagged pilins, lysed in a high-pressure cell disrupter and the supernatants were applied to GSH Sepharose Fast Flow affinity resin. *E. coli* expressing only GST was included as a negative control. Elution fractions were separated by SDS-PAGE and the upper gel part was stained with Coomassie brilliant blue and the lower gel part was immunoblotted with antisera specific for ComGD, ComGE or ComGG. **(B, C)** Co-immunoprecipitation (IP) of minor pilins co-expressed in *E. coli*. The bacterial pellet from cells co-expressing the soluble domains of ComGD, ComGE, ComGF and ComGG was lysed, centrifuged and the supernatant was immunoprecipitated using anti-ComGG antibodies coupled to Protein G Dynabeads™ (+). Uncoupled beads (-) were included as a negative control. IP samples were separated by SDS-PAGE followed by **(B)** Coomassie brilliant blue staining and **(C)** immunoblotting with antibodies against ComGD, ComGE, ComGF and ComGG.

Others showed previously that minor pilins/pseudopilins can interact *via* their globular C-terminal domains (Yanez et al., 2008; Douzi et al., 2009; Cisneros et al., 2012; Mann et al., 2013). To investigate possible interactions between the pneumococcal minor pilins, we used an *in vitro* co-purification assay with affinity-tagged minor pilins in *E. coli*. The C-terminal domains of ComGC, ComGD, ComGE, ComGF and ComGG were cloned into pGEX4T1 to express them as N-terminal GST-tagged fusion proteins or cloned into pET21a to express untagged recombinant pilins. GST-tagged and untagged proteins were expressed individually. *E. coli* pellets expressing each untagged pilin were then mixed with *E. coli* pellets expressing each GST-tagged pilin. Cells were lysed and the pilins were purified from the lysate. As control, pellets expressing each untagged pilin were mixed with pellets expressing GST alone. All purified GST-tagged pilins were detected by Coomassie Brilliant Blue staining (**Figure 3A**, upper gels). The GST-tagged minor pilins were slightly unstable and multiple bands that could correspond to degradation products or other contaminants were observed. We then probed the eluate fractions for ComGD, ComGE or ComGG by immunoblotting and found that all three minor pilins were pulled-down by GST-D, GST-E, GST-F and GST-G, suggesting that they could interact with each other and themselves (**Figure 3A**, lower gels). Untagged soluble ComGF could not be stably produced in *E. coli* and was therefore not tested in this assay. None of the minor pilin globular domains interacted detectably with GST-ComGC.

Based on these results, we hypothesized that all four minor pilins likely interact and that ComGF requires the presence of other minor pilins for its stability. To address this question, we co-expressed the C-terminal domains of ComGD, ComGE, ComGF, and ComGG in *E. coli* and performed co-immunoprecipitation with ComGG antiserum. When expressed together in *E. coli*, distinct bands in the range of 5-15 kDa corresponding to the sizes of the soluble domains of the individual minor pilins were visible in the eluate after Coomassie staining (**Figure 3B**), and all four minor pilins were detected by immunoblotting (**Figure 3C**). These data suggest that 1) ComGF is stable in the presence of ComGD, ComGE and ComGG, and 2) ComGF can either directly interact with ComGG, consistent with the pull-down results, or 3) through another minor pilin.

### ComGD, ComGF and ComGG Are Present in Sheared Pili

**Figure 4:**
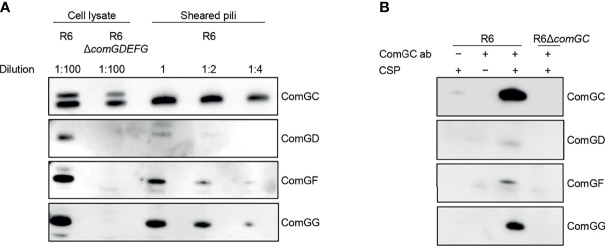
Localization of ComGD, ComGF and ComGG. **(A)** Whole-cell lysate (diluted 1:100) and the sheared fraction (2-fold serial dilutions as indicated) from WT R6 were analysed by immunoblotting with antisera specific for ComGC, ComGD, ComGF and ComGG. The lysate from a strain lacking all four minor pilins (R6*ΔcomGDEFG*) was included as a negative control and indicates the specificity of detection. **(B)** Co-immunoprecipitation of sheared pili from WT R6 strain using anti-ComGC antibodies (ComGC ab). As negative controls a non-induced culture (-CSP), a no antibody control (-ComGC ab) and a strain lacking the major pilin (*R6ΔcomGC*) were included. IP samples were probed for the presence of ComGC, ComGD, ComGF and ComGG by immunoblotting.

Next, we investigated the abundance of ComGD, ComGF and ComGG in pneumococcal lysates and sheared pili fractions of the WT R6 strain by immunoblotting (**Figure 4A**). All three minor pilins were detected in R6 lysates but were absent in a mutant lacking *comGDEFG*. ComGD, ComGF and ComGG were also detected in sheared pili fractions with ComGG being the most abundant. As expected, the proportion of ComGD, ComGF or ComGG was much lower than the proportion of the major pilin ComGC (**Figure 4A**). To further examine whether ComGD, ComGF and ComGG are associated with competence pili, we performed co-immunoprecipitation on sheared pili fractions using ComGC antisera. All three minor pilins tested were found in sheared pili of CSP-induced cultures, suggesting that they are part of the pilus, and were absent in non-induced cultures, and in a strain lacking ComGC (**Figure 4B**). Minor amounts of ComGC were detectable in the CSP induced no antibody condition, probably due to unspecific binding to Protein A sepharose.

### ComGF Is Incorporated Throughout the Competence Pilus

**Figure 5:**
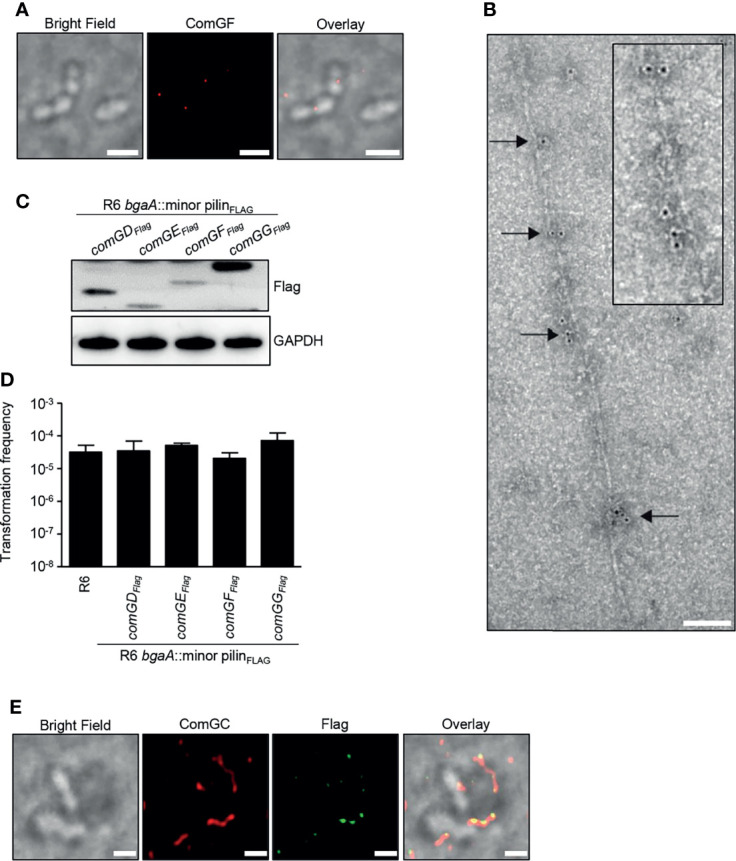
Visualization of the minor pilin ComGF. **(A)** BF and IF microscopy of competent WT R6 bacteria stained with anti-ComGF antibodies (red). Scale bars represent 2 µm. **(B)** Immunogold electron microscopy of competent WT R6 bacteria. Electron micrographs were stained with anti-ComGF antibodies and secondary antibodies conjugated to 6-nm gold particles. Black arrows indicate gold particles incorporated throughout the pilus filament. The inset (black box) shows an enlargement of the immunogold-labelled pilus. Scale bar represents 100 nm. **(C)** Bacterial whole cell lysates of R6 strains encoding an extra copy of C-terminal Flag-tagged *comGD*, *comGE*, *comGF* or *comGG* in the *bgaA* locus were analysed by immunoblotting using Flag-antiserum. GAPDH was used as loading control. **(D)** Transformation frequency of WT R6 and R6 strains encoding an extra copy of C-terminal Flag-tagged minor pilins. The error bars represent the standard deviation of a minimum of three independent experiments (n=3). **(E)** Co-localization of ComGF_Flag_ and ComGC visualized by IF microscopy. Competent bacteria ectopically expressing Flag-tagged ComGF (R6 *bgaA*::*comGF_Flag_*) were labelled with primary antibodies specific for ComGC (red) and anti-Flag antibodies (green). Bacteria were visualized by BF microscopy. Scale bars correspond to 1 µm.

Because ComGD, ComGF and ComGG were detected in sheared pili fractions, we also performed immunofluorescence microscopy to examine the localization of these minor pilins. While we failed to detect any signal in competent WT bacteria stained with ComGD or ComGG antisera, specific ComGF signal was detected in competent bacteria stained with ComGF antisera (**Figure 5A**). To confirm that the ComGF signal is associated with the competence pilus, we performed immunogold electron microscopy with ComGF antisera and observed labeling of ComGF sporadically throughout the competence pilus, indicating that the minor pilin ComGF may be incorporated into the pilus filament (**Figure 5B**).

To further investigate the possible localization of ComGD, ComGE and ComGG, strains expressing a Flag-tagged minor pilin were generated. Direct insertion of the sequence encoding the Flag tag in the *comG* operon was not possible because all minor pilins are overlapping. For that reason, an additional copy of *comGD*, *comGE*, *comGF* or *comGG* encoding a C-terminal Flag-tag was integrated ectopically at the *bgaA* locus under the control of the native CSP-inducible promoter. Expression of the Flag-tagged minor pilins was confirmed by immunoblotting using anti-Flag antibodies (**Figure 5C**), and the transformation frequency of these strains was similar to WT levels (**Figure 5D**). Consistent with the electron microscopy results, ComGF, detected with mouse anti-Flag antibodies, co-localized with the competence pilus stained with rabbit anti-ComGC serum (**Figure 5E**). In contrast, ComGD-Flag, ComGE-Flag or ComGG-Flag could not be detected despite repeated attempts.

### ComGD_E5A_ and ComGE_E5A_ Affect Competence Pilus Formation and Function

**Figure 6:**
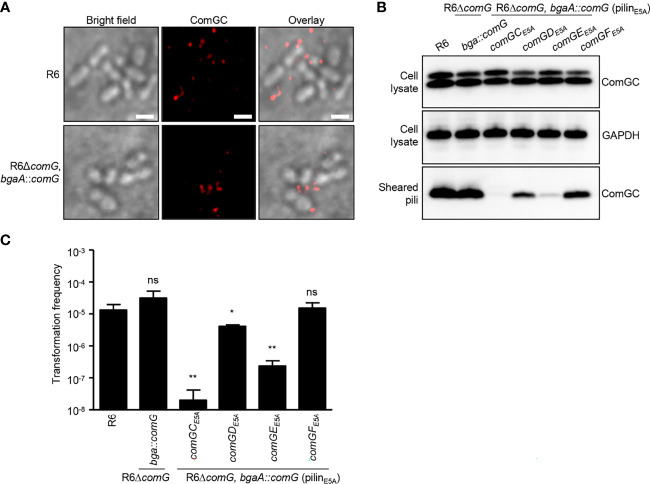
E5A mutations affect minor pilin function of ComGD and ComGE. **(A)** Visualization of competence-induced pili in WT R6 and R6*ΔcomG, bgaA*::*comG* complemented strain by IF microscopy. Bacteria were visualized by bright field microscopy and competence pili were labelled with antibodies specific for ComGC (red). Scale bars represent 2 µm. **(B)** ComGC was detected by immunoblotting in bacterial whole cell lysates and sheared pili of E5A mutant strains (*comGC_E5A_*, *comGD_E5A_*, *comGE_E5A_* or *comGF_E5A_*). The WT R6 strain and the complemented mutant strain in which the native *comG* operon was expressed ectopically (R6*ΔcomG*, *bgaA*::*comG*) were included as positive controls. GAPDH was used as a loading control. **(C)** Transformation frequency of WT R6 strain, R6*ΔcomG*, *bgaA*::*comG *complemented mutant and E5A mutant strains. The error bar represents the standard deviation of a minimum of three independent experiments. A t-test was used for statistical analysis: *p ≤ 0.05 and **p ≤ 0.01, ns, no significant difference (*C_E5A_ p* = 0.0043*; D_E5A_ p* = 0.0237 and *E_E5A_ p* = 0.0047).

A conserved feature among type IV pilins is the invariant E5 residue in position 5 after the prepilin cleavage site, and substitution of E5 in the major pilin ComGC by valine abolishes competence pilus assembly and function (Laurenceau et al., 2013). To gain insight into the role of the E5 residue present in ComGD, ComGE and ComGF, we generated E5 variants in these minor pilins and tested their ability to assemble pili and to take up DNA. Given the difficulties of directly manipulating the native *comG* operon, we reintroduced the *comG* operon including its promoter ectopically at the *bgaA* locus using a modified version of the integration vector pJVW25, and subsequently deleted the native operon. Before we constructed similar integration vectors with minor pilin E5 mutations, we verified that our control strain expressing *comG* ectopically in *bgaA*, and deficient in its native *comG* operon (R6*ΔcomG, bgaA*::*comG*), was functional. Indeed, using immunofluorescence microscopy, we were able to detect pili on the surface of competent R6*ΔcomG, bgaA*::*comG* bacteria (**Figure 6A**) and more importantly, this strain was transformable (**Figure 6C**). Encouraged by these results, E5A substitutions were constructed in *comGD*, *comGE* and *comGF* and the strains were tested for their ability to produce pili. ComGC was detected in similar amounts in whole-cell lysates of all mutant strains carrying E5A, the *comG* control strain and the WT R6 strain (**Figure 6B**). Consistent with previous findings for ComGC_E5V_ (Laurenceau et al., 2013), ComGC was absent in sheared pili in a ComGC_E5A_ strain (**Figure 6B**). The levels of ComGC in the sheared pili fractions of the strains with E5A substitution in the minor pilins showed varying amounts of ComGC compared to the control strain and were overall reduced in the ComGD_E5A_ and ComGE_E5A_ strain (**Figure 6B**). Surprisingly, pilus formation in the presence of the ComGE_E5A_ variant was nearly abolished, suggesting a dominant negative effect of this amino acid substitution. The transformation frequency of those strains correlated well with the amount of ComGC present in sheared pili, and the ComGD_E5A_ and ComGE_E5A_ strain had significantly reduced transformation rates. These results reveal that E5 is necessary for two of the three minor pilins in which it is conserved, and suggests that ComGD, and especially ComGE, rely more heavily on their N-terminal α-helix interactions.

## Discussion

The pneumococcal competence pilus belongs to the T4P family and is essential for DNA uptake of this pathogen. The major pilin ComGC forms the backbone of the competence pilus, however, the contribution of the minor pilins, ComGD, ComGE, ComGF and ComGG in pilus biogenesis and function remained unclear.

In the present study, we show that mutant strains deficient in ComGDEFG, ComGEFG, ComGFG and ComGG are non-piliated and non-transformable. This suggests that ComGG is essential for pilus assembly, and that the minor pilin(s) ComGD, ComGDE and ComGDEF are not sufficient for piliation. Moreover, neither individual minor pilins, nor ComGDEF were able to restore piliation in a *ΔcomGDEFG* background, and functional competence pili were only assembled in presence of all minor pilins. *Bacillus subtilis* expresses a homologous *comG* operon and consistent with our findings, all minor pilins in *B. subtilis* are required for pilus assembly and transformation (Chung and Dubnau, 1998; Chen et al., 2006). Strikingly, also T2SSs contain five pilins (one major and four minor pseudopilins), and all minor pseudopilins are required for efficient PulA secretion by the *Klebsiella oxytoca* T2SS (Possot et al., 2000). The finding that all minor pilins are required for competence pilus assembly suggests that they are interdependent and that their translation must be tightly orchestrated to form a stable and functional complex. Studies in *E. coli* that demonstrate a link between the operon gene order and efficient co-translational assembly of protein complexes, would support this assumption (Shieh et al., 2015; Wells et al., 2016). Thus, deletion of any of the minor pilin genes would hamper the formation of a stable minor pilin complex and thereby abolish competence pilus formation. Interestingly, in *P. aeruginosa* it was shown that a specific stoichiometric ratio among the minor pilins was important for pilus function, and loss or overexpression of minor pilins impaired the twitching motility (Giltner et al., 2010).

A main finding in this study is that all four minor pilins can directly interact with each other and likely form a minor pilin complex. It is important to note that we used the soluble domains of the pneumococcal minor pilins to test putative interactions and therefore might have missed other interactions involving the hydrophobic domains. The observation that the soluble domain of ComGC did not interact with soluble ComGG, whereas fullength ComGC and ComGG interacted in the BACTH assay, confirmed this assessment. In line with our results, others reported that the soluble domain of the major pilin in *B. subtilis* (ComGC) and in *Pseudomonas aeruginosa* (XcpT) did not interact with minor pilins lacking the hydrophobic domain (Douzi et al., 2009; Mann et al., 2013).

Another important finding is the intriguing role of ComGG. Our results showed that fullength ComGG and ComGC directly interact, further supported by two observations: (I) all deletion mutants lacking ComGG showed reduced levels of the major pilin ComGC and (II) ComGG alone is able to stabilize ComGC when ectopically expressed in a strain lacking all four minor pilins. Several examples of minor subunits that provide a link to the major subunit have been identified both in T4P and T2SS biogenesis. In *P. aeruginosa* it was proposed that the minor pilin complex PilVWXE together with the large, non-pilin PilY1, form an assembly initiation complex, while FimU couples this complex to the major pilin subunit PilA (Nguyen et al., 2015). In contrast to ComGG, FimU is incorporated into pili in the absence of the other minor pilins (Nguyen et al., 2015). The structure of FimU strongly resembles the minor pseudopilin GspH in *E. coli*, and the GspH pseudopilin homolog in *P. aeruginosa*, XcpU, was proposed to link the pseudopilin XcpVWX complex to the major pseudopilin XcpT (Douzi et al., 2009; Nguyen et al., 2015). In the *Klebsiella* T2SS, the minor pilin PulH, a XcpU homologue, links the priming complex composed of PulI, PulJ and PulK to the major pseudopilin PulG (Cisneros et al., 2012).

ComGG is also important for the stabilization of the other minor pilins, and in a ComGG deficient strain, no ComGF and less ComGD was detected. Similar observations on mutually dependent minor pilins have been reported in other bacteria (Winther-Larsen et al., 2005; Mann et al., 2013). Intriguingly, ComGG is the last minor pilin in the *comG* operon and the only pneumococcal minor pilin that lacks the conserved E5 residue. It was previously speculated that ComGG could form the competence pilus tip (Piepenbrink and Sundberg, 2016). This was based on the observation that E5 is involved in interactions with the N-terminus of the previously incorporated pilin into the growing pseudopilus fiber, and hence would be dispensable for the first pilin subunit integrated into the pilus (Korotkov et al., 2012). *E. coli* GspK is an example of such a protein and was shown to form a trimeric complex with GspI and GspJ, presumably at the tip of the pseudopilus (Korotkov and Hol, 2008). We find ComGD, ComGF and ComGG in the sheared pili fraction of competent *S. pneumoniae* and by co-immunoprecipitation of sheared pili. However, we were not able to detect ComGG by immunofluorescence microscopy or electron microscopy in the competence pilus. This could simply be due to its low abundance, especially if there is only one of each per pilus. It could also reflect variability in antibody affinities for the different pilins or different accessibilities of epitopes for staining, hence does not rule out the potential localization of ComGG at the pilus tip.

Notably, our immunofluorescence and electron microscopy data suggest that ComGF is incorporated throughout the competence pilus, which has also been demonstrated for minor pilins in *P. aeruginosa* and *Clostridium difficile* T4P (Giltner et al., 2010; Piepenbrink et al., 2015). Since ComGF was not stable in the absence of ComGG nor the other minor pilins, it is tempting to speculate that the entire minor pilin complex becomes incorporated into the pilus. It could also mean that ComGF has additional pilus-related functions besides its role in the minor pilin complex and becomes stabilized differently when built into the ComGC fiber. Because the minor pilin mutant expressing ComGF_E5A_ was not impaired in pilus formation and function, interactions with ComGF are presumably E5-independent. In contrast, competent bacteria expressing ComGD_E5A_ or ComGE_E5A_ variants showed reduced levels of ComGC in the sheared pili fraction and reduced transformation frequencies compared to the WT R6 strain, revealing a critical role for E5 in ComGD and ComGE in competence pilus biogenesis.

Minor pseudopilins in Gram-negative bacteria have been proposed as initiators and terminators of pseudopilus assembly. In the “initiator” scenario, pseudopilus assembly takes place beneath the minor pseudopilin complex, which would consequently end up at the tip of the pilus forming a cap (Korotkov et al., 2012). Alternatively, the minor pseudopilin complex remains in the inner-membrane and integrates major pseudopilin subunits from the base, thereby controlling/terminating pseudopilus elongation (Douzi et al., 2009). A more dynamic role for PilY1 and the minor pilins in *Myxococcus xanthus* was recently reported. In this model, PilY1 and the minor pilins form a priming complex to initiate T4P extension. The complex locates at the tip of the pilus and at the same time functions as terminator of pilus retraction (Treuner-Lange et al., 2020). It is interesting to consider whether the pneumococcal minor pilins may have a similar function since the competence pilus was recently shown to be a dynamic structure that is able to retract for efficient DNA uptake (Lam et al., 2021).

Because all pneumococcal minor pilins are required for pilus formation and given their ability to interact with each other, we propose that the pneumococcal minor pilins form a complex and that ComGG couples this complex to the major pilin ComGC. Based on our results, the function of the pneumococcal minor pilin complex appears to be primarily related to initiation of competence pilus assembly and/or elongation. The precise interaction network and the molecular events leading to the formation of such a putative minor pilin complex and it´s localization will require further studies.

## Data Availability Statement

The original contributions presented in the study are included in the article/supplementary material, further inquiries can be directed to the corresponding authors.

## Author Contributions

VO, SM and BHN designed the study. VO, MSA, AvE, and SM performed the experiments. VO, SM, and BHN wrote the manuscript. All authors contributed to writing. All authors contributed to the article and approved the submitted version.

## Conflict of Interest

The authors declare that the research was conducted in the absence of any commercial or financial relationships that could be construed as a potential conflict of interest.

## Publisher’s Note

All claims expressed in this article are solely those of the authors and do not necessarily represent those of their affiliated organizations, or those of the publisher, the editors and the reviewers. Any product that may be evaluated in this article, or claim that may be made by its manufacturer, is not guaranteed or endorsed by the publisher.
